# THE EFFECT OF ACTIGRAPHY MEASURED PHYSICAL ACTIVITY ON EXECUTIVE FUNCTION IN OLDER ADULTS

**DOI:** 10.1093/geroni/igac059.2387

**Published:** 2022-12-20

**Authors:** Pilar Thangwaritorn, Hilary Hicks, Alex Laffer, Genna Losinski, Kayla Meyer, Elizabeth Tran, Keri Cox, Amber Watts

**Affiliations:** University of Kansas, LAWRENCE, Kansas, United States; University of Kansas, Lawrence, Kansas, United States; University of Kansas, Lawrencee, Kansas, United States; University of Kansas, Lawrence, Kansas, United States; University of Kansas Alzheimer’s Disease Research Center, Kansas City, Kansas, United States; University of Kansas Alzheimer’s Disease Research Center, Kansas City, Kansas, United States; University of Kansas Alzheimer’s Disease Research Center, Kansas City, Kansas, United States; University of Kansas, Lawrence, Kansas, United States

## Abstract

Executive function (i.e., decision making, self-control, planning) is important for facilitating independent living in older adults. Physical activity may preserve executive function, but previous research has demonstrated sex differences in both physical activity and executive function among older adults. Few studies have investigated sex differences in the association between the two. We examined associations between objectively measured physical activity and executive function with attention to sex differences. We recruited N = 204 participants (Mage =71, SD=6.36; 57% women) with (n=47) and without (n=157) Alzheimer’s disease from the University of Kansas Alzheimer’s Disease Research Center. We used wrist-worn accelerometers (Actigraph GT9X) to measure physical activity 24 hours a day for 7 days in a free-living environment. We categorized physical activity as moderate to vigorous (MVPA) based on the Montoye (2020) Adult Vector Magnitude cut-points. We evaluated sex differences in the association between executive function and MVPA using multiple regression with an interaction term, adjusting for age, education, and dementia status. We used a composite score to combine tests of executive function (Digit Symbol Substitution, Stroop Interference, Trail making Part B, and Verbal Fluency). Results indicated, older age and lower education were associated with lower executive function scores (β=-2.12, p < 0.001; B=2.13, p < .05). In contrast to previous research, we did not find evidence for sex differences in the MVPA, executive function, nor the association between the two in our sample. Future research should investigate whether individualized exercise-based interventions and treatment between men and women may differentially benefit cognitive function.

